# Dr. Adelbert G. Gumaer

**Published:** 1909-10

**Authors:** 


Dr. Adelbert G. Gumaer, formerly of Buffalo, died August 1, 1909 in Santa Monica, Cal. He was well known in this city, where he had practised his profession for many years. Dr. Gumaer went to California for his health last fall. He was 59 years old when he died.

After receiving a B. S. degree at Michigan University, Dr. Gumaer went to the University of Pennsylvania for his degree of M. D.

While still in college Dr. Gumaer married Miss Sarah E. Lumley, who died in 1888, leaving three children, Florence I., Adelbert G., and Percy W. In 1890, he married Miss Josephine Adams. There was one daughter from this union. Mrs. Gumaer, her daughter Carolyn and her stepdaughter Florence survive. They will continue to live in Santa Monica.

